# Vitamin D-Related Information Exposure, Attitudes, and Practices Among Prostate and Breast Cancer Survivors in Poland: Implications for Patient–Physician Communication

**DOI:** 10.3390/nu18030427

**Published:** 2026-01-28

**Authors:** Anita Mikołajczyk, Karolina Kaczmarczyk, Mateusz Mikołajczyk

**Affiliations:** 1 Department of Psychology and Sociology of Health and Public Health, Collegium Medicum, University of Warmia and Mazury in Olsztyn, Warszawska Str. 30, 10-082 Olsztyn, Poland; 2 Student Scientific Group of Public Health Research, Collegium Medicum, University of Warmia and Mazury in Olsztyn, Warszawska Str. 30, 10-082 Olsztyn, Poland; kaczmarczykakarolina@gmail.com; 3 Masovian Dental Center Ltd., Nowy Zjazd Str. 1, 00-301 Warsaw, Poland

**Keywords:** vitamin D, 25-hydroxyvitamin D, 25(OH)D, breast cancer survivor, prostate cancer survivor, patient–physician communication

## Abstract

Introduction: The potential impact of vitamin D deficiency on cancer risk and oncological management remains under investigation. The study aimed to analyse vitamin D information exposure, attitudes, and practices, including the use of vitamin D and other supplements as well as serum 25-hydroxyvitamin D [(25(OH)D)] testing, among breast cancer and prostate cancer survivors in the context of patient–physician communication. Material and methods: This cross-sectional survey included 145 breast and prostate cancer survivors (mean age 62.2 ± 9.9 years) who participated using an original, validated questionnaire. Results: Between cancer diagnosis and survey completion, more than 52% of respondents reported vitamin D supplementation, and nearly 65% declared using supplements other than vitamin D in relation to their cancer. However, only 17.3% of respondents had been instructed by their physician to take vitamin D, and only 5.5% had been ordered by their physician to have their serum 25(OH)D levels tested. The majority of respondents (94%) perceived the need for physicians to routinely order vitamin D level tests for patients diagnosed with cancer. Only 39.5% of vitamin D users and 7.5% of other supplement users stated that their oncology care physicians knew about their supplement intake. The most common reason for patients not to inform their physician about taking vitamin D and/or other supplements was that the physician did not ask about this issue. Almost all aspects analysed showed greater health information exposure and better communication skills with physicians among women than among men. Conclusions: It appears reasonable to improve physician–patient communication and broaden consideration of patients’ needs, which suggests a direction for further studies on the role of routine 25(OH)D testing in the standard care of patients with breast and prostate cancer.

## 1. Introduction

The incidence and mortality rates of cancers are a significant public health problem both in Poland and globally, accounting for 3 out of 10 premature deaths [1]. The scale of this phenomenon is confirmed by the systematic increase in the number of diagnosed cases, with forecasts indicating that by 2050, the number of cancer incidences may increase by 77% in comparison to 2022 [2].

In an international context, Poland is among the countries with the highest cancer mortality rates [3]. As regards men, the most commonly diagnosed cancer is prostate cancer, with an upward trend in mortality rates observed since 2004. In women, however, the most common cancer is breast cancer, whose incidence has been rising steadily for over 50 years [4,5]. Globally, breast cancer is the second most commonly diagnosed cancer after lung cancer, while prostate cancer is one of the five most common cancers among men [2]. According to 2022 data provided by the International Agency for Research on Cancer (IARC), it is estimated that, globally, one in twelve women and one in nine men die from cancer [2]. The forecast for incidence and mortality in Poland indicates a continuation of long-term trends with an upward trend for prostate cancer in men, and breast cancer in women [6].

In the period from 2015 to 2020, the mortality rate from prostate cancer in Poland increased by 18%, whereas in most EU countries, it decreased by 7.1% [7]. This higher mortality may be partly related to systemic factors in Polish cancer care, including limited healthcare financing, problems with distribution and access to medical specialists and specialized hospitals, and restricted access to modern therapies, which can delay diagnosis and reduce treatment effectiveness [4,8]. Despite advances in breast cancer treatment, Poland still presents a high mortality rate, similar to that in countries with significantly higher incidence rates [3]. In view of the high costs of oncological treatment (chemotherapy, radiotherapy, surgery), there is a constant search for supportive strategies that could prevent or enhance the effectiveness of treatment [9]. One such measure is research into the relationship between carcinogenic processes and vitamin D, including its deficiencies.

Scientific literature increasingly indicates that vitamin D deficiency may be associated with an increased risk of mortality in human populations [10,11]. Attention is also drawn to the potential anti-cancer effects of vitamin D, demonstrated, e.g., in cell culture studies and pre-clinical models [12]. Some studies suggest that there is a link between 25-hydroxyvitamin D [(25(OH)D], a commonly used laboratory marker of vitamin D status in the body) and the risk of developing breast cancer in premenopausal women and prostate cancer in men [13]. This relationship is explained by the role of vitamin D in regulating numerous genes responsible for the proliferation, apoptosis, differentiation, and communication of cancer and immune cells [14,15,16]. Vitamin D is metabolised in two stages: initially in the liver (where 25(OH)D_3_, or calcidiol, is produced) and then in the kidneys (where the active form 1,25(OH)_2_D_3_, or calcitriol, is produced) [17]. The active form of vitamin D binds to nuclear VDRs (Vitamin D Receptors), which modulate the expression of numerous genes involved in cell proliferation, differentiation, and apoptosis [18,19]. These receptors are found in most cells of the body, including cancer cells, which demonstrates the broad spectrum of action of vitamin D. The binding of calcitriol to VDRs may block the cell cycle of cancer cells and regulate the expression of the prostaglandin synthesis pathway [20]. In the context of breast cancer, calcitriol has been shown to inhibit the activity of cancer-associated fibroblasts, e.g., by reducing the production of chemokines, which are important for the migration of immune cells [21]. In addition, vitamin D metabolites support the functioning of the innate immunity, which may play an important role in cancer prevention in genetically predisposed individuals [22]. Vitamin D affects the immune system by modulating molecular pathways that regulate the differentiation and activation of immune cells, which in turn control cancer cells [19].

Vitamin D deficiency is a widespread global issue. Sources of vitamin D include both endogenous (skin synthesis) and exogenous (dietary and supplemental) sources [17]. Epidemiological data from Poland indicate that more than half of the population has an average 25(OH)D level below 20 ng/mL. Almost 25% of the subjects achieve suboptimal values (20–30 ng/mL), and only approximately 10% meet the accepted standards [15]. Vitamin D deficiency affects both the general population and those who are sick. In a study conducted abroad on oncology patients, nearly half had 25(OH)D levels below 20 ng/mL [23]. Values below this reference threshold of 20 ng/mL are classified as a deficiency requiring supplementation to 30–100 ng/mL [24]. In Poland, vitamin D fortification is limited; although some products (including margarines and infant/children’s modified milk) are enriched with vitamin D, overall dietary intake remains insufficient for most of the population [24,25]. Although a potential link between vitamin D and cancer has been reported, data on vitamin D supplementation, serum level monitoring, and patient–physician communication among Polish breast and prostate cancer survivors remain scarce.

This study aimed to assess vitamin D information exposure, attitudes, and practices (including the use of vitamin D and other supplements as well as serum 25(OH)D testing) among breast and prostate cancer survivors through a cross-sectional survey. Selected attitudes and practices regarding vitamin D supplementation and blood testing were analysed in the context of cancer patient–physician communication. In this study, it was assumed that gaps exist in patient–physician communication concerning vitamin D supplementation among breast and prostate cancer survivors.

## 2. Materials and Methods

### 2.1. Definition and Clarifications

A cancer survivor is defined as a person from the moment of diagnosis onward, for as long as they live. This period can encompass active treatment, post-treatment follow-up, and long-term health maintenance or late-effect treatment. A cancer survivor is considered a patient when receiving care from a physician. In the current study, breast or prostate cancer survivors were selected according to the established inclusion and exclusion criteria.

“Breast and prostate cancer survivors” used interchangeably with “breast cancer survivors and prostate cancer survivors,” refers to two participant groups: women who survived breast cancer and men who survived prostate cancer.

In Poland, cancer care involves a wide range of medical specialists, including medical oncologists, surgical oncologists, radiation oncologists, urologists, haematologists, pathologists, and primary care physicians. For example, prostate and breast cancer are treated by a range of specialists, including medical oncologists, surgical oncologists, urologists, and radiation oncologists, depending on the stage and type of cancer. Accordingly, the term “oncology care physician” is used in the study survey to refer to a medical specialist involved in the care of cancer patients, including cancer treatment and/or care for cancer survivors.

Vitamin D deficiency is defined as serum 25-hydroxyvitamin D [25(OH)D] levels < 50 nmol/L (20 ng/mL) [15,24].

### 2.2. Participants, the Inclusion and Exclusion Criteria

This study was a cross-sectional survey. The study was carried out among 145 prostate and breast cancer survivors from August 2023 to December 2024. Seventy-four women (breast cancer survivors) and 71 men (prostate cancer survivors) were included in the survey. All participants belonged to the Polish branches of patient organizations supporting patients with breast and prostate cancer.

The inclusion criteria for patients to participate in the study were as follows: age ≥ 18 years; in a period of no less than two years and no more than five years after the confirmed diagnosis of breast or prostate cancer. The study assumed that the assessment of practices related to vitamin D supplementation and laboratory testing of vitamin D levels should also cover a period of at least two and no more than five years after the cancer diagnosis.

The exclusion criteria for patients to participate in the study were as follows: patients with other cancer diagnoses besides breast cancer and prostate cancer; patients with recurrent and metastatic cancer; patients surveyed in a period of less than two years or more than five years after the confirmed diagnosis of breast cancer or prostate cancer.

It should be noted that observed differences between women and men may partly reflect differences in cancer type, as all female participants were breast cancer survivors and all male participants were prostate cancer survivors (Table 1).

### 2.3. Survey Questionnaire

The study employed a proprietary, self-administered Polish questionnaire developed specifically for this research. Its construction was preceded by an analysis of previously published literature on the subject, which informed the survey’s content and structure. According to Polish regulations, anonymous, non-interventional survey research that does not collect personal or clinical data that enables patient identification is not classified as a medical experiment and therefore does not require approval from a bioethics committee. The study was carried out in accordance with ethical standards and in line with the principles of the Declaration of Helsinki, and all participants provided informed consent.

#### 2.3.1. Questionnaire Design

The questionnaire applied in this research was developed in accordance with the general methodological recommendations described by Boparai et al. (2019) [26]. It included 13 core closed-ended questions and an additional set of 8 socio-demographic items. The English version of the questionnaire is available in the Supplementary Materials.

The study used a questionnaire, administered online or in person by an interviewer. All participants received information about the study, including details on voluntary, anonymous participation. No incentives were provided to the individuals. Some individuals declined participation, and those who met the exclusion criteria described in Section 2.2 were deemed ineligible.

Google Forms was used to create online questionnaires, which were automatically hosted via a unique URL. A unique study ID ensured the confidentiality of all self-reported data. There was little interest in participating in the online surveys. Furthermore, the data collected via the online questionnaire (Google Forms) did not meet the study’s criteria. Consequently, data obtained from online surveys were excluded from the analysis.

Finally, data for analysis were collected during interviews using traditional face-to-face questionnaires administered by an interviewer who asked each participant questions directly. Consequently, 74 breast cancer survivors and 71 prostate cancer survivors answered the questionnaire questions and were enrolled in the study after meeting the study criteria. Their responses were analysed using the chosen statistical software.

#### 2.3.2. Reliability, Comprehensibility and Acceptability

The questionnaire was validated on a sample of 52 respondents. Analyses were performed to assess its comprehensibility, acceptability, and reliability. To evaluate the repeatability of answers to the questions, the percentage agreement was estimated. The same questionnaire was administered to the same group of participants in a two-week interval, and the degree of repeatability of responses was estimated by Kappa-Cohen’s coefficient. This coefficient ranges from 0 to 1, with values interpreted as follows: 0.81–1.00 indicating almost perfect agreement, 0.61–0.80 substantial agreement, 0.41–0.60 moderate agreement, 0.21–0.40 fair agreement, and below 0.21 slight agreement [27]. The reliability assessment revealed very good repeatability for all key items, with Kappa values ranging from 0.97 to 1.00.

The comprehensibility and acceptability of the questionnaire were evaluated by two public health experts using a semi-structured interview. All participants reported that the questionnaire’s format was appropriate, the font size was clear, and the questions were easy to understand. The majority of respondents (98%) considered the survey length to be adequate. None of the participants indicated that any questions were difficult to answer or that they preferred not to respond to certain items. The average completion time was 6.6 min. Additionally, all respondents indicated that completing the questionnaire prompted them to seek information about the potential relationship between vitamin D deficiency and cancer.

### 2.4. Statistical Analysis

The chi-square test was used to assess differences in characteristics, information exposure, attitudes, and practices regarding vitamin D use, patient–physician communication regarding vitamin D supplementation, between breast and prostate cancer survivors, and according to demographic factors. The difference in age between males and females and patient–physician communication regarding vitamin D supplementation was estimated using the t-student test. A *p*-value < 0.05 was considered to be significant. The data analysis was conducted using Statistica (data analysis software), version 13 [StatSoft Polska Sp. z o.o., 2024; www.statsoft.pl (accessed on 22 January 2026)].

## 3. Results

### 3.1. Characteristics of Participants

The study involved 145 individuals, including 74 (51%) female breast cancer survivors and 71 (49%) male prostate cancer survivors. The mean age was 62 years, with 59 years in the women’s group and 66 years in the men’s group. The women participating in the study were not only younger but were also more frequently employed than the men. Among men, retired employees were the largest group (59.2%), whereas among women, professionally active individuals were the largest group (52.7%). Furthermore, secondary education was more prevalent among women (41.9%), whereas higher education was more prevalent among men (57.7%). More than half of respondents (51.7%) lived in cities with populations over 100,000. The majority of respondents (78.6%) declared their marital status to be “married”. It should be noted that observed differences between women and men may partly reflect differences in cancer type, as all female participants were breast cancer survivors and all male participants were prostate cancer survivors (Table 1).

### 3.2. Information Exposure, Attitudes, and Practices Regarding Vitamin D, Including the Use of Vitamin D and Other Supplements, as Well as Serum 25(OH)D Testing Among Breast and Prostate Cancer Survivors

Less than 19% of respondents had heard about the link between vitamin D deficiency and an increased risk of cancer development, with women significantly more frequently than men (29.7% of women vs. 7.0% of men; *p* < 0.001) declaring information exposure of this link. Professionally active individuals were also significantly more likely than retired respondents to report having heard such claims (28.1% vs. 9.6%; *p* = 0.005) (Table S1). Slightly over 26% of respondents reported having heard claims that abnormal vitamin D levels can impact the course of cancer, and, again, women were significantly more likely than men (44.6% vs. 7.0%; *p* < 0.001) to report having heard such claims (Table 2). These claims were most frequently reported by respondents with higher education (38.2%), followed by those with secondary education (21.1%), whereas markedly lower rates were observed among respondents with vocational (15.4%) and primary education (0%). They were also more commonly reported by professionally active individuals than by retired respondents (42.2% vs. 12.3%; *p* < 0.001) (Table S1).

Despite low information exposure of the link between vitamin D and oncogenesis, slightly over half (52.4%) of the study participants reported supplementing vitamin D between their cancer diagnosis and the survey and, in connection with this supplementation, had not discontinued any recommended therapeutic treatment. It should be stressed that supplementation was significantly more common among women than men (73.0% vs. 31.0%; *p* < 0.001), with the most common dose among women being 4000 IU, whereas among men it was 2000 IU. Among respondents, 35.1% supplemented vitamin D on their own initiative, or on the initiative of their loved ones. It should be noted that only 17.3% of respondents were instructed by their physician to take vitamin D. Women had more frequently than men been instructed by their physicians to supplement vitamin D (32.5% vs. 1.4%; *p* < 0.001). Among the group of women surveyed, vitamin D supplementation was prescribed by oncologists and family physicians. In contrast, among the men surveyed, the physician recommending vitamin D supplementation was a urologist. Almost 65% of respondents of both sexes (60.8% of women and 69.0% of men) declared that they were taking supplements other than vitamin D in connection with their cancer. It should be emphasised that these individuals declared that they had not discontinued any recommended therapeutic treatment in connection with taking these supplements (Table 2). Vitamin D supplementation since cancer diagnosis was most frequent among respondents with higher education (66.2%), followed by those with secondary education (63.2%), whereas markedly lower rates were observed among participants with primary (23.1%) and vocational education (15.4%). The use of supplements other than vitamin D was also most frequent among respondents with higher education (86.8%), followed by those with secondary education (57.9%), whereas substantially lower rates were observed among participants with vocational (34.6%) and primary education (30.8%). Individuals with secondary education most frequently reported deciding to start vitamin D supplementation on their own (31.6%), followed by those with higher education (29.4%), whereas markedly lower rates were observed among respondents with primary (7.7%) and vocational education (0%). Self-prescribed vitamin D supplementation was also significantly more common among professionally active respondents compared with retired individuals (31.5% vs. 14.1%; *p* = 0.01) (Table S1).

The majority (84.1%) of respondents, i.e., breast cancer and prostate cancer survivors, did not have their 25(OH)D levels tested—76% of women and 93% of men (*p* = 0.04) did not have their 25(OH)D levels tested between the date of their cancer diagnosis and the date of the survey. In contrast, 10.4% of respondents had their vitamin D levels tested on their own initiative or at the initiative of a loved one. Only 5.5% of respondents had their vitamin D levels tested following their physicians’ orders—for women, these were medical oncologists and endocrinologists, and for men, urologists. Physicians significantly more frequently ordered vitamin D level tests for women than for men (9.4% vs. 1.4%; *p* = 0.04) (Table 2). Respondents who were bachelor/maiden most frequently reported arranging vitamin D level testing on their own (28.6%), followed by those who were divorced (12.5%) and married (6.2%), whereas no widower/widow respondents reported self-arranged (Table S1). Despite the low number of physicians who ordered 25(OH)D level testing, the vast majority of respondents (93.8%) perceived the need for physicians to routinely recommend vitamin D level testing for oncology patients (Table 2). Education level, place of residence, professional activity, and marital status did not have a significant impact on responses regarding the perceived need for physicians to order such tests routinely (Table S1).

### 3.3. Patient–Physician Communication and the Patient’s Attitude Towards the Obligation to Provide Information to the Physician Among Breast Cancer and Prostate Cancer Survivors

Although a relatively small percentage of study participants (4.8%) believed that they should not inform their oncology care physician about taking vitamin D and other supplements in connection with cancer, in addition to the respondents who were convinced of the obligation to inform their physician (55.9%), there was also a group of individuals (39.3%) who made their decision to inform their oncology care physician dependent on their belief of the physician’s views on supplementation (Table 2). Furthermore, significant differences were observed in terms of sex. It should be noted that the observed differences between women and men may reflect not only sex but also differences in age, cancer type, and treatment approaches, since all female participants were breast cancer survivors and all male participants were prostate cancer survivors. Nevertheless, we observed significant differences between women and men regarding whether oncology patients should inform their physician about taking vitamin D and other supplements. Significantly more women than men (89.2% vs. 21.2%; *p* < 0.001) believed that oncology patients should inform their oncology care physician about taking vitamin D and other supplements in connection with their cancer. However, men significantly more frequently than women (74.6% vs. 5.4%; *p* < 0.001) based their decision to inform their oncology care physician about their knowledge of the physician’s views on supplementation, claiming that if they sensed that the physician was unfavourable towards supplementation, it was better not to mention the issue to him/her (Table 2). Beyond differences related to sex and cancer type, other sociodemographic characteristics were also associated with respondents’ views on whether oncology care physicians should be informed about patients’ use of vitamin D and other supplements. Respondents with primary, secondary, and vocational education most frequently believed that physicians should be informed (76.9%, 76.3%, and 73.1%, respectively), whereas this view was less common among respondents with higher education (33.8%). Moreover, respondents who were widower/widow most frequently expressed this view (87.5%), followed by bachelor/maiden (85.7%), whereas married (56.1%) and divorced (25.0%) respondents reported it less frequently. The ambivalent response, indicating that the decision to inform the physician depended on the physician’s views on supplementation, was selected significantly more often by respondents with higher education (61.8%) than by those with lower education levels (Table S1).

**Figure 1:**
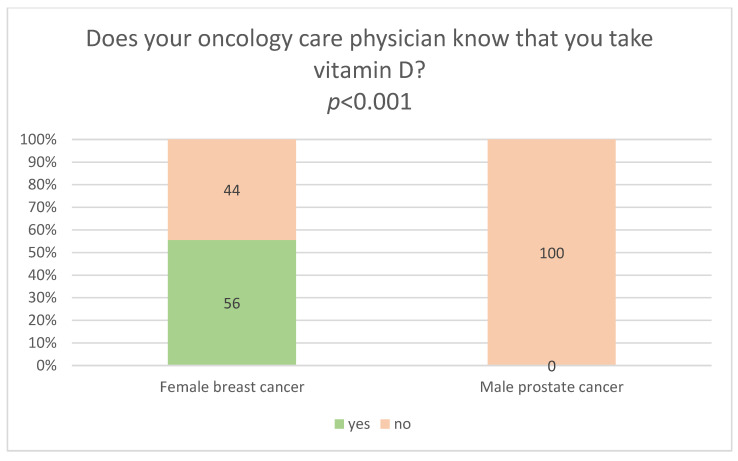
Relationship between the sex (female breast cancer survivors and male prostate cancer survivors) taking vitamin D, and their oncology care physician’s knowledge of vitamin D supplementation.

Among the individuals taking vitamin D from the date of their cancer diagnosis until the date of the survey, only 39.5% said that their oncology care physicians knew about their vitamin D use. This percentage represents the sum of respondents who had informed their physician about it (6.6%), or those whose physician had recommended taking vitamin D (31.6%), or those whose physician had asked whether they take vitamin D (1.3%) (Table 3). Professionally active respondents were significantly more likely than retired respondents to report that their oncology care physicians knew about their vitamin D use (63.4% vs. 30%; *p* < 0.001). Respondents who reported that their oncology care physicians knew about their vitamin D use were also significantly younger than those who reported no such awareness (mean age 58.7 ± 7.9 vs. 65.7 ± 10.1 years; *p* = 0.002) (Table S2). Among the individuals taking vitamin D, women were significantly more likely than men to report that their oncology care physicians knew about their use (56% vs. 0%; *p* < 0.001) (Figure 1). Among women, the most common reason for their physicians’ knowledge of supplementation was that the physicians had recommended that their patients take this vitamin. Among the men surveyed, none had informed their oncology care physician about their vitamin D use, most often citing that their physician had not asked them about it. Similarly, among women supplementing vitamin D and not informing their physician about its use, the most frequently cited reason for not informing oncology care physicians about their vitamin D use was that the physician did not ask about it (Table 3).

**Figure 2:**
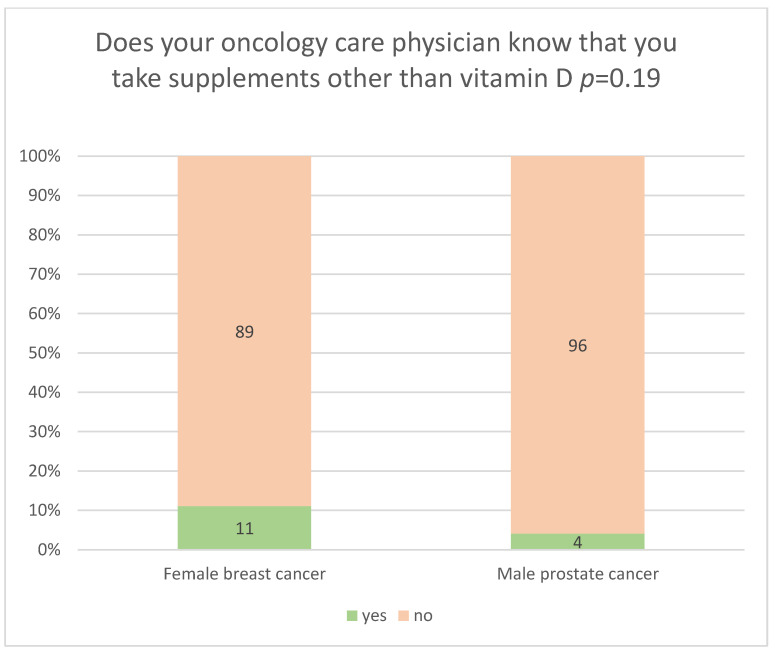
Relationship between the sex (female breast cancer survivors and male prostate cancer survivors) using supplements other than vitamin D, and their oncology care physician’s knowledge of the use of supplements other than vitamin D.

Among the individuals taking supplements other than vitamin D in connection with their cancer, only 7.5% said that their oncology care physicians knew about their use of supplements other than vitamin D. As in the case of vitamin D, women significantly more frequently than men informed their oncology care physicians about their use of supplements other than vitamin D (11% vs. 4%, *p* = 0.19) (Figure 2). Nevertheless, regardless of sex, the majority (92.5%) of respondents using supplements other than vitamin D did not inform their physicians about their use, most often because the physician did not ask about it, and because they were afraid that the physician would forbid them from using supplements if he or she found out about it. Interestingly, men, unlike women, were also afraid of being ridiculed by their physician (Table 4). Education level, place of residence, professional activity, and marital status did not have a significant impact on respondents’ declarations regarding whether their oncology care physician was aware of their use of supplements other than vitamin D (Table S2).

## 4. Discussion

This is the first nation-wide study that the authors are aware of to report on selected trends in vitamin D supplementation and blood testing in the context of patient–physician communication.

Epidemiological data from Poland and other countries confirm the growing problem of vitamin D deficiency in the general population, especially among high-risk groups, including oncology patients [14,15,16,28]. In view of the fact that vitamin D deficiency is observed in up to 90% of the Polish population, and considering the rationality of managing the budget appropriations derived from health insurance funds, the Ministry of Health and many Polish experts believe that there are no grounds for introducing the determination of 25(OH)D levels to the catalogue of reimbursed tests performed to the order of a primary care physician. A recent study conducted in Poland indicates the need to determine the concentration of 25(OH)D in the elderly population taking vitamin D [29]. The present study indicates that the majority (84.1%) of respondents did not have their 25(OH)D levels tested from the date of their cancer diagnosis until the date of the survey. Significantly more men (93%) than women (76%) had not had their 25(OH)D levels tested between the date of their cancer diagnosis and the date of the survey. Although only 5.5% of respondents had their vitamin D levels tested following their physicians’ orders, 10.4% had them tested on their own initiative or at the initiative of a loved one, paying for the test with their own funds (Table 2).

Bearing in mind the high mortality rate due to cancer, and the long-term forecasts of an increase in the incidence and mortality rates in Poland, with an upward trend for prostate cancer in men and breast cancer in women [6], it is reasonable to search for and implement supportive strategies that could prevent or enhance the effectiveness of treatment. Therefore, it appears that one such measure could be regular monitoring of 25(OH)D levels in oncology patients and high-risk individuals, and possible supplementation for deficiencies. Although some literature reviews [30] report that routine determination of 25(OH)D levels in healthy populations does not appear to be medically necessary, attention should be paid to oncology patients and the relationship between vitamin D and the carcinogenesis process. Systematic reviews and meta-analyses [31,32] suggest the need for further research into both the potential role of vitamin D in cancer prevention and therapy, and the association between low serum 25(OH)D levels and an increased risk of breast and prostate cancer development. Vitamin D, through its active form calcitriol, binds to the vitamin D receptor (VDR) in various cells, including cancer cells, regulating genes involved in proliferation, apoptosis, and differentiation. Calcitriol also modulates immune responses and may inhibit cancer-associated fibroblasts, chemokine production, and angiogenesis, potentially contributing to its anticancer effects [19,21]. Nevertheless, the literature also warns of the potential effects of excessive vitamin D supplementation without proper monitoring, as elevated concentrations can, e.g., adversely affect bone metabolism [33]. Research, discussion, and consideration of the above issues may lead to the dissemination of 25(OH)D monitoring in clinical practice and, consequently, provide an inexpensive and effective tool to support cancer prevention and treatment.

The results of the present study indicate a low, but sex-dependent frequency of 25(OH)D level determination in oncology patients: women diagnosed with breast cancer had more than three times as frequently as men with prostate cancer had their vitamin D levels tested between the date of cancer diagnosis and the date of the survey (Table 2). A similar sex-related pattern was observed in Canada, where women in Manitoba had their 25(OH)D levels tested twice as frequently as men. In addition, respondents in the above study expressed the need for physicians to order vitamin D level testing [30]. Similarly, in the present study, breast cancer and prostate cancer survivors perceived the need for physicians to routinely order tests for vitamin D deficiency in oncology patients (Table 2). Therefore, it appears that paying attention to the needs of oncology patients as regards vitamin D level testing, and in-depth knowledge about the relationship between vitamin D and the carcinogenic process, should encourage physicians to increase the frequency of vitamin D level testing.

Due to the widespread vitamin D deficiency observed in various age and social groups, this problem is becoming a global health challenge [34]. Due to geographical factors, Polish inhabitants are particularly vulnerable to insufficient vitamin D synthesis in the skin; therefore, knowledge of the principles of supplementation with this vitamin is very important [35]. The results of the present study show that the majority of respondents (82.7%) did not receive any recommendations from their physician as regards vitamin D supplementation, and as many as 95% of participants had not had their 25(OH)D levels tests ordered by a physician from the date of cancer diagnosis until the date of the survey (Table 2). It seems the reasons for this situation lie with the physicians themselves. A study by Zgliczyński et al. (2021) [36], conducted among Polish physicians participating in specialisation courses at the Postgraduate Medical Education Centre in 2019, showed that 61% of them did not recommend routine vitamin D supplementation to their patients. Moreover, most of the physicians surveyed did not regularly take vitamin D, and only 25% monitored their serum 25(OH)D levels. An assessment of physicians’ knowledge of vitamin D found it to be average; unfortunately, only 1.4% of the physicians surveyed answered all questions correctly. The results varied depending on variables such as sex, medical specialisation, and frequency of recommending supplementation to patients [36]. This phenomenon is not limited to Poland. In the United Kingdom, a country with limited insolation, physicians have limited knowledge of vitamin D deficiency prevention, despite the availability of official guidelines [37]. The results of a study conducted in Sudan showed a low level of knowledge among physicians about vitamin D deficiency and its treatment, with almost half of the physicians presenting a negative attitude towards supplementation, which resulted in the lack of action on their part to counteract vitamin D deficiencies in the population [38]. A study conducted among physicians and medical students in Saudi Arabia revealed that fewer than 1/3 of physicians, and fewer than 1/5 of interns were able to correctly identify the recommended dose of vitamin D [39].

The literature also indicates that introducing thematic training in oncology among medical students significantly improves their knowledge in this area [40]. It can therefore be assumed that systematic training on the relationship between vitamin D and the carcinogenic process, especially at higher levels of medical education, can bring measurable benefits. In light of the above, it is imperative to urgently educate physicians, including oncologists, about the role of vitamin D in cancer and ways to address the growing problem of vitamin D deficiency in the population. It is also necessary to increase physicians’ involvement in discussions with patients about supplementation and its potential benefits. The validity of this need is confirmed by the results of the present study. This issue is particularly important in the case of men diagnosed with prostate cancer. Vitamin D supplementation was prescribed significantly less frequently to men (1.4%) than to women (32.5%). As many as 93% of the men surveyed reported not having heard claims about a link between vitamin D deficiency and an increased risk of cancer development, or about the potential impact of abnormal vitamin D levels on the course of the disease (Table 2).

Effective communication between a physician and a patient is a key tool in diagnosis, treatment and health monitoring. Proper communication enables the physician to obtain reliable information about the patient’s physical and mental condition, while allowing the patient to understand the treatment process better and actively participate in it. Friendly, empathetic communication has a positive effect on patients’ health, increases their satisfaction with medical care, and reduces anxiety associated with treatment [41,42].

In the present study, respondents most frequently reported not informing their physician about their vitamin D supplementation or use of supplements other than vitamin D because the physician did not ask them about it (Table 3 and Table 4). This may indicate poor quality in physician-patient communication.

Women significantly more frequently than men communicated with their oncology care physician about their use of vitamin D (56% vs. 0%) (Figure 1) or supplements other than vitamin D (11% vs. 4%) (Figure 2). This shows that male patients may find it more difficult to establish open communication with medical personnel. In addition, female breast cancer survivors (89.2%) significantly more frequently than male prostate cancer survivors (21.2%) believed they should inform their oncology care physician about their use of vitamin D and supplements based on, e.g., other vitamins, herbs, or mushrooms (Table 2). This trend is also confirmed by other studies, which show that women more frequently report a positive attitude towards conversations with their physician and greater openness to cooperation [43]. They are also more convinced that effective communication contributes to the success of therapy. Good relationships with physicians motivate patients, particularly those with chronic diseases, to adhere to therapeutic recommendations [44].

Physician-patient communication is of utmost importance in oncological care. Dietary supplements used by patients are particularly relevant in this regard. Although the efficacy of many supplements is low or clinically insignificant, and the expected effects often fail to materialise [45], public confidence in dietary supplements remains high [46], and the supplement industry is growing rapidly, partly due to the society’s desire for self-medication, longer life expectancy, and the prevention of lifestyle diseases [47]. In the context of cancer, the sense of cognitive uncertainty plays an important role, characterised by intense anxiety related to the unpredictability of the further course of the disease, recurrence and survival prognosis [48]. This condition is among the key psychosocial stressors in patients with cancer [49]. In people with reduced mental well-being, self-medication, including the use of broadly defined CAM (Complementary and Alternative Medicine), can serve a psychological function, namely, give a sense of control, reduce anxiety and improve mood. Research shows that up to 91% of oncology patients in Poland have used at least one form of CAM as a complementary therapy [50]. Similar results were noted, e.g., in Saudi Arabia, where 70% of oncology patients reported using CAM [51].

In the present study, 64.8% of respondents reported using supplements other than vitamin D in connection with cancer, e.g., preparations based on herbs, mushrooms, amygdalin, or vitamin C (Table 2). Individuals using these supplements did not discontinue any recommended medical treatment. However, it is important to note that the majority (92.5%) of individuals taking supplements did not inform their physicians, citing that their physicians did not ask about it as the main reason. In addition, respondents mentioned fear of being forbidden from using supplements by their physician, and men also feared being ridiculed by their physician (Table 4).

Although women (89.2%) significantly more frequently than men (21.2%) took the view that the oncology care physician should be informed by the patient about the use of vitamin D and/or other supplements, the present study also shows that respondents’ attitudes towards informing their physician about the use of supplements depended on their subjective assessment of the physician’s position on supplementation. Respondents had a less positive attitude towards informing the physician if they perceived him/her to be sceptical or critical of supplements. Importantly, men (74.6%) were significantly more likely than women (5.4%) to have the attitude that the decision to inform their physician about the use of vitamin D and/or other supplements depended on their perception of the physician’s views on supplementation, whether favourable or not, and if they sensed a negative attitude towards supplementation, it was better not to disclose this information (Table 2).

Some reasons for not informing physicians about the use of supplements by patients may result from the belief that the physician may be sceptical about this fact, and patients using supplements feel that their mental well-being has improved, which they do not want to lose not only by discontinuing the use of supplements, but also, often subconsciously, do not want that criticism of their chosen supplementation would lead to a deterioration in their mental well-being. Studies show that for many patients, the primary motivation for using supplements is to regain control over their health, improve their mood, and reduce feelings of helplessness [52]. Buckner et al. (2018) indicate that more than half of Canadian oncology patients who use CAM do so out of a sense of obligation to “do everything possible” [53]. However, French patients most often reported improving their mental well-being as their main motive for using CAM [54]. Polish hospital patients used CAM mainly to enhance the efficacy of conventional treatment and to alleviate therapy-related side effects [55].

Therefore, when communicating with an oncology patient, the physician must be aware of the relationship between the patient’s use of a particular supplement and their mental state. This will help the patient engage in an honest conversation. This is particularly important given that some supplements used by patients, especially herbal supplements, may interact with anticancer drugs, including chemotherapeutics and molecularly targeted drugs, thereby affecting therapy effectiveness [56]. Supplements can intensify side effects or weaken the effects of cancer treatment, especially when patients abandon conventional therapy in favour of alternative medicine [57,58]. It is obvious that the use of CAM as an alternative to, rather than a supplement to, cancer treatment is associated with an increased risk of death [59].

Although various institutions in Poland, including the Chamber of Physicians and Dentists and the Patient Ombudsman, are leading information initiatives to promote brochures on the need for patients to inform their physicians about the medicines and supplements they take, a study [60] indicates that the disclosure of information remains a serious challenge for healthcare providers, and is largely determined by the nature of patient-provider communication during consultation and perceptions of the provider’s knowledge of CAM.

Other reasons for poor communication between the physician and a patient include a lack of training in interpersonal skills and insufficient use of non-verbal communication [61]. Patients notice a lack of eye contact with the physician, often due to the physician’s need to complete medical documentation during the visit. Non-verbal communication, i.e., facial expressions, gestures and body posture, can also be important for the quality of the physician-patient relationship [62]. It is also worth noting that the duration of a medical consultation in Poland and other countries is often strictly limited. Physicians consciously control the dynamics of the conversation in order to conform to the imposed time frame [63]. In a study conducted in the Persian Gulf countries, most physicians reported a lack of time as the primary obstacle to effective communication with their patients [64,65].

In view of the above, it is essential to strengthen physicians’ communication skills, not only through formal education during medical studies, but also through postgraduate training. Developing interpersonal communication skills can significantly improve the quality of physician-patient relationships, increase patients’ involvement in the treatment process, and enhance treatment effectiveness, particularly in chronic diseases and cancers. It is therefore extremely important that oncology patients inform their physicians of all relevant information, especially their psychosocial needs, including those related to dietary supplements and CAM. Open and empathetic communication built on trust can enhance the physician’s ability to identify potential interactions between the prescribed treatment and any supplements and alternative therapies the patient is using. Furthermore, it allows for a responsive approach to the patient’s mental health needs, guiding them through the treatment process without relying on unverified and potentially hazardous practices.

Interestingly, in the case of vitamin D use, as with supplements, most patients did not inform their physicians, because the physician did not ask about it; the other reasons were rather different. The respondents, when asked why they did not inform their physician about their use of vitamin D, were not afraid of being ridiculed by their physician, or of being forbidden from using vitamin D, as was the case with supplements other than vitamin D. However, they believed that the reason for not informing their physician about their use of vitamin D was that they considered this type of information to be irrelevant, or they justified it by saying that they had forgotten to (Table 3). It is difficult to explain clearly what caused this attitude. Perhaps cancer patients are aware that vitamin D is registered in Poland not only as a supplement, but also as a medicine, so they do not have such strong fears of being ridiculed by their physician, or of being forbidden from using it, as is the case with supplements other than vitamin D, which are not registered as medicines. The authors regard the absence of a question on this issue in the survey as a limitation of the study, and, at the same time, perhaps an idea for further research.

The present study was also limited by a small, though sufficiently appropriate, participant group, a direct consequence of its stringent exclusion criteria. Furthermore, the survey assumed the use of the term “vitamin D” without distinguishing among different types of vitamin D supplements, i.e., D_3_ (cholecalciferol) and D_2_ (ergocalciferol). However, since in Poland the preferred and commercially available medical products contain vitamin D_3_, it can be assumed that for respondents, the term “vitamin D” is the same as “cholecalciferol”. Another limitation is that it was not possible to verify whether the responses regarding physicians’ orders to supplement vitamin D or to test serum 25(OH)D levels matched the patients’ medical records. Additionally, although we collected information on vitamin D supplementation, we did not collect detailed data on the duration of each dose, changes in dosing over time, timing, clinical indication, or monitoring of supplementation. Collecting this type of detailed information could be the focus of future research. It should also be noted that observed differences between women and men may partly reflect differences in cancer type and treatment approaches, as all female participants were breast cancer survivors and all male participants were prostate cancer survivors. Therefore, sex-related differences reported in this study might also be interpreted in the context of cancer type and the associated care pathways.

Despite these limitations, the study identified key findings regarding vitamin D-related information exposure, attitudes, and practices among prostate and breast cancer survivors in Poland, providing insights into problems in patient–physician communication.

### Synthesis of Key Points to Conclude All Obtained and Discussed Results:

(1) Most participants of the study had no information exposure regarding of the link between vitamin D deficiency and the risk of cancer development (81% of respondents), and that abnormal levels of vitamin D in the body can impact the course of cancer (74% of respondents).
(2) Over 52% of cancer survivors surveyed took vitamin D supplements from the date of their cancer diagnosis until the date of the survey.
(3) Nearly 65% of cancer survivors surveyed declared that they were taking supplements other than vitamin D in connection with their cancer.
(4) Both respondents taking vitamin D in connection with their cancer, and respondents taking supplements other than vitamin D, did not discontinue any recommended medical therapies.
(5) Only 17.3% of respondents were prescribed vitamin D by a physician between the date of their cancer diagnosis and the date of the survey.
(6) The majority (84.1%) of respondents did not have their blood 25(OH)D levels tested between the date of their cancer diagnosis and the date of the survey.
(7) Only 5.5% of respondents had their blood 25(OH)D levels tests ordered by their physicians between the date of their cancer diagnosis and the date of the survey.
(8) The majority of respondents (94%) perceived the need for physicians to routinely order vitamin D level tests for patients diagnosed with cancer.
(9) Among vitamin D users, only 39.5% reported that their oncology care physicians knew about their vitamin D intake. Only 7.5% of respondents taking supplements other than vitamin D stated that their oncology care physicians knew about their use of supplements other than vitamin D. The others most frequently indicated that the reason for not informing their physician about taking supplements was that the physician did not ask them about this issue.
(10) Sex plays a significant role in communication styles, with men and women often exhibiting different preferences and approaches:
  - Women with breast cancer (73%) more frequently than men with prostate cancer (31%) took vitamin D. Physicians recommended vitamin D supplementation to women more often than to men (32.5% vs. 1.4%). Physicians significantly more frequently ordered serum vitamin D testing for women than for men (9.4% vs. 1.4%).
  - Women significantly more frequently than men informed their oncology care physicians about the use of vitamin D and other supplements. Among individuals taking vitamin D, women were significantly more likely than men to report that their oncology care physicians knew about its use (56% vs. 0%). Similarly, women were significantly more likely than men to inform their physicians about the use of supplements other than vitamin D (11% vs. 4%). Only men, unlike women, indicated that one reason for not informing their oncology care physicians about the use of supplements other than vitamin D was fear of being ridiculed by the physician.
  - Women (89.2%) significantly more frequently than men (21.2%) believed that they should inform their physician about taking vitamin D and/or other supplements. In contrast, men (74.6%) significantly more frequently than women (5.4%) based their decision to inform their physician about taking vitamin D and/or supplements other than vitamin D on their perception of the physician’s views on supplementation, claiming that if they sensed that the physician was unfavourable towards supplementation, it was better not to mention the issue to him/her. Women (89.2%) were significantly more likely than men (21.2%) to hold the attitude that physicians should be informed about the use of vitamin D and/or other supplements. Men (74.6%), however, were significantly more likely than women (5.4%), to have the attitude that their decision to inform the physician about the use of vitamin D and/or other supplements depended on perception of the physician’s views on supplementation, stating that if they sensed the physician held a negative attitude toward supplementation, it was better not to disclose it.

## 5. Conclusions

Analysis of the results of the survey conducted among breast cancer survivors and prostate cancer survivors indicates their limited information exposure regarding the relationship between vitamin D deficiency and the process of carcinogenesis, as well as a low percentage of respondents who have been prescribed vitamin D by their physicians. Nevertheless, 52% of those surveyed had used vitamin D between their cancer diagnosis and the survey. Although 94.5% of participants had not been ordered by their physician to take a 25(OH)D test, the majority of respondents (93.8%) expressed the need for physicians to routinely order tests for vitamin D deficiency in patients diagnosed with cancer. Most respondents also admitted that they did not inform their oncology care physicians about taking vitamin D and other supplements. One of the most common reasons was the physician’s lack of questions about this issue. These findings highlight existing gaps in patient–physician communication. However, women (breast cancer survivors) were more willing to discuss this topic, whereas men (prostate cancer survivors) more frequently feared a negative reaction from their physician, including being ridiculed, which may have influenced their attitude and level of openness. Men more frequently than women breast cancer survivors based their decision to inform their oncology care physician about taking vitamin D and/or supplements other than vitamin D on their subjective perception of the physician’s views on supplementation, claiming that if they sensed that the physician was unfavourable towards supplementation, it was better not to mention it to him/her.

Improving the quality of physician–patient communication, with particular attention to the needs of oncology patients, including those related to dietary supplements and complementary and alternative medicine, is an important consideration. This suggests a direction for further studies on the role of routine 25(OH)D testing in the standard care of patients with breast and prostate cancer.

## Figures and Tables

**Table 1:** Characteristics of participants.

	All		Women Breast Cancer Survivors		Men Prostate Cancer Survivors		
	n = 145	%	n = 74	%	n = 71	%	*p*
Age: average ± SD	62.2 ± 9.9		58.7 ± 10.3		65.8 ± 8.2		<0.001
Educational level							<0.001
Primary	13	9.0	6	8.1	7	9.9	
Basic vocational	26	17.9	10	13.5	16	22.5	
Secondary	38	26.2	31	41.9	7	9.9	
Higher	68	46.9	27	36.5	41	57.7	
Place of residence:							0.26
Village	27	18.6	13	17.6	14	19.7	
City with a population of up to 100,000	43	29.7	18	24.3	25	35.2	
City with a population of over 100,000	75	51.7	43	58.1	32	45.1	
Professional activity/respondents could tick more than one answer/							0.31
Student	1	0.7	0	0.0	1	1.4	
I am still professionally active	64	44.1	39	52.7	25	35.2	
At present, on sick leave or receiving rehabilitation allowance	2	1.4	1	1.4	1	1.4	
Unemployed	3	2.1	2	2.7	1	1.4	
Retired employee	73	50.3	31	41.8	42	59.2	
Disability pensioner	2	1.4	1	1.4	1	1.4	
Marital status							0.02
Married	114	78.6	53	71.6	61	85.9	
Bachelor/maiden	7	4.9	7	9.5	0	0.0	
Divorced	16	11.0	8	10.8	8	11.3	
Widower/widow	8	5.5	6	8.1	2	2.8	

**Table 2:** Information exposure, attitudes, and practices regarding vitamin D, including the use of vitamin D and other supplements, as well as serum 25(OH)D testing among breast cancer and prostate cancer survivors.

	All		Women Breast Cancer Survivors		Men Prostate Cancer Survivors		*p*
	n = 145	%	n = 74	%	n = 71	%	
**Information exposure**							
**Have you heard claims that vitamin D deficiency may increase the risk of cancer?**							<0.001
Yes	27	18.6	22	29.7	5	7.0	
No	118	81.4	52	70.3	66	93.0	
**Have you heard claims that abnormal vitamin D levels in the body can affect the course of cancer?**							<0.001
Yes	38	26.2	33	44.6	5	7.0	
No	107	73.8	41	55.4	66	93.0	
**Practices**							
**Have you been supplementing vitamin D since your cancer diagnosis?**							<0.001
No	69	47.6	20	27.0	49	69.0	
Yes, and in connection with vitamin D supplementation, I have not discontinued any recommended therapeutic treatment	76	52.4	54	73.0	22	31.0	
Yes, and in connection with vitamin D supplementation, I have discontinued the recommended therapeutic treatment, recognising that vitamin D was more efficient in the treatment of my cancer than the medical recommendations	0	0.0	0	0.0	0	0.0	
**Have you used/are you using supplements other than vitamin D in connection with your cancer, e.g.,** **herb-, mushroom-, amygdalin- or vitamin C-based supplements?**							0.30
No	51	35.2	29	39.2	22	31.0	
Yes, and in connection with their supplementation, I have not discontinued any recommended therapeutic treatment	94	64.8	45	60.8	49	69.0	
Yes, and in connection with their supplementation, I have discontinued the recommended therapeutic treatment, recognising that supplements are more efficient in the treatment of my cancer than the medical recommendations	0	0.0	0	0.0	0	0.0	
**Who has recommended vitamin D supplementation to you since your cancer diagnosis?**							<0.001
No one	69	47.6	20	27.0	49	69.0	
I have recommended it to myself	33	22.7	20	27.0	13	18.3	
A physician	25	17.3	24	32.5	1	1.4	
A family member or an acquaintance who is not a physician	18	12.4	10	13.5	8	11.3	
**Who has recommended that you have your vitamin D levels tested since your cancer diagnosis?**							0.04
No one	122	84.1	56	75.7	66	93.0	
I have recommended it to myself	11	7.6	9	12.2	2	2.8	
A physician	8	5.5	7	9.4	1	1.4	
A family member or an acquaintance who is not a physician	4	2.8	2	2.7	2	2.8	
**Attitudes**							
**Do you perceive a need for physicians to routinely order tests for vitamin D deficiency in patients diagnosed with cancer?**							0.23
Yes	136	93.8	68	91.9	68	95.8	
No	1	0.7	0	0.0	1	1.4	
I have no opinion on this matter	8	5.5	6	8.1	2	2.8	
**Do you think that your oncology care physician should be informed about your use of vitamin D and/or supplements based on other vitamins, herbs or mushrooms in connection with your cancer?**							<0.001
Yes	81	55.9	66	89.2	15	21.2	
No	7	4.8	4	5.4	3	4.2	
It is difficult to answer “yes” or “no” because it depends on the physician’s views on this type of supplementation, and if one senses that the physician is not in favour of supplementation, it is better not to mention it	57	39.3	4	5.4	53	74.6	

**Table 3:** Physician-patient communication and vitamin D supplementation by the patient.

Does Your Oncology Care Physician Know That You Take Vitamin D? *(Respondents Could Tick More Than One Answer)*	Answers from People Taking Vitamin D						
	All		Women Breast Cancer Survivors		Men Prostate Cancer Survivors		*p*
	n = 76	%	n = 54	%	n = 22	%	
Yes, I have informed the physician about it, and he/she has not forbidden me from using vitamin D	5	6.6	5	9.3	0	0.0	NA
Yes, I have informed the physician about it, and he/she has forbidden me from using vitamin D	0	0.0	0	0.0	0	0.0	NA
Yes, the physician knows about it, because he/she has ordered me to take vitamin D her/himself	24	31.6	24	44.4	0	0.0	NA
Yes, the physician knows about it, because he/she has asked me whether I take vitamin D, and has not forbidden me from continuing to use it	1	1.3	1	1.9	0	0.0	NA
Yes, the physician knows about it, because he/she has asked me whether I take vitamin D, and has forbidden me from continuing to use it	0	0.0	0	0.0	0	0.0	NA
No, I have not informed the physician about it, because he/she has not asked me about it	46	60.5	24	44.4	22	100.0	NA
No, I have not informed the physician about it, because I have forgotten to tell him/her about it	28	36.8	11	20.4	17	77.3	<0.001
No, I have not informed the physician about it, because I considered it to be irrelevant	34	44.73	17	31.5	17	77.3	<0.001
No, I have not informed the physician about it, because I thought he/she might ridicule me	0	0.0	0	0.0	0	0.0	NA
No, I have not informed the physician about it, because I thought he/she might forbid me from using it	0	0.0	0	0.0	0	0.0	NA
Yes, because … (other reason)	0	0.0	0	0.0	0	0.0	NA
No, because … (other reason)	0	0.0	0	0.0	0	0.0	NA

NA—not available.

**Table 4:** Physician-patient communication and the patient’s use of supplements other than vitamin D.

Does Your Oncology Care Physician Know That You Take Supplements Other Than Vitamin D? *(Respondents Could Tick More Than One Answer)*	Answers from People Taking Supplements Other Than Vitamin D						
	All		Women Breast Cancer Survivors		Men Prostate Cancer Survivors		*p*
	n = 94	%	n = 45	%	n = 49	%	
Yes, I have informed the physician about it, and he/she has not forbidden me from using supplements	4	4.3	2	4.4	2	4.1	0.93
Yes, I have informed the physician about it, and he/she has forbidden me from using supplements	0	0.0	0	0.0	0	0.0	NA
Yes, the physician knows about it, because he/she has ordered me to take supplements	2	2.1	2	4.4	0	0.0	NA
Yes, the physician knows about it, because he/she has asked me whether I take supplements, and has not forbidden me from continuing to use them	1	1.1	1	2.2	0	0.0	NA
Yes, the physician knows about it, because he/she has asked me whether I take supplements, and has forbidden me from continuing to use them	0	0.0	0	0.0	0	0.0	NA
No, I have not informed the physician about it, because he/she has not asked me about it	87	92.6	40	88.9	47	95.9	0.20
No, I have not informed the physician about it, because I have forgotten to tell him/her about it	2	2.1	2	4.4	0	0.0	NA
No, I have not informed the physician about it, because I considered it to be irrelevant	2	2.1	2	4.4	0	0.0	NA
No, I have not informed the physician about it, because I thought he/she might ridicule me	41	43.6	0	0.0	41	83.7	NA
No, I have not informed the physician about it, because I thought he/she might forbid me from using these supplements	56	59.6	15	33.3	41	83.7	<0.001
Yes, because … (other reason)	0	0.0	0	0.0	0	0.0	NA
No, because … (other reason)	0	0.0	0	0.0	0	0.0	NA

NA—not available.

## Data Availability

All data are presented in this study, and details may be made available upon request from the corresponding author. The data consist of anonymous responses from respondents’ surveys and contain no personally identifiable information.

## Supplementary Materials

The following supporting information can be downloaded at: https://www.mdpi.com/article/10.3390/nu18030427/s1, File S1. Questionnaire—English version; Table S1. Information exposure, attitudes, and practices regarding vitamin D, including the use of vitamin D and other supplements, as well as serum 25(OH)D testing among breast cancer and prostate cancer survivors by the socio-demographic data. Table S2. Socio-demographic characteristics of breast cancer and prostate cancer survivors according to their oncology care physician’s awareness of their vitamin D supplementation and their use of supplements other than vitamin D.

## References

[1] Bray F., Laversanne M., Weiderpass E., Soerjomataram I. (2021). The Ever-increasing Importance of Cancer as a Leading Cause of Premature Death Worldwide. Cancer.

[2] Bray F., Laversanne M., Sung H., Ferlay J., Siegel R.L., Soerjomataram I., Jemal A. (2024). Global Cancer Statistics 2022: GLOBOCAN Estimates of Incidence and Mortality Worldwide for 36 Cancers in 185 Countries. CA Cancer J. Clin..

[3] Dyba T., Randi G., Bray F., Martos C., Giusti F., Nicholson N., Gavin A., Flego M., Neamtiu L., Dimitrova N. (2021). The European Cancer Burden in 2020: Incidence and Mortality Estimates for 40 Countries and 25 Major Cancers. Eur. J. Cancer.

[4] Didkowska J.A., Wojciechowska U., Barańska K., Miklewska M., Michałek I., Olasek P. (2023). Nowotwory Złośliwe w Polsce w 2021 Roku [Cancer in Poland in 2021]. Krajowy Rejestr Nowotworów [Polish National Cancer Registry].

[5] Didkowska J., Wojciechowska U., Michalek I.M., Caetano Dos Santos F.L. (2022). Cancer Incidence and Mortality in Poland in 2019. Sci. Rep..

[6] Didkowska J., Barańska K., Miklewska M.J., Wojciechowska U. (2024). Cancer Incidence and Mortality in Poland in 2023. Nowotw. J. Oncol..

[7] Carioli G., Bertuccio P., Boffetta P., Levi F., La Vecchia C., Negri E., Malvezzi M. (2020). European Cancer Mortality Predictions for the Year 2020 with a Focus on Prostate Cancer. Ann. Oncol..

[8] Gliniewicz A., Dudek-Godeau D., Bielska-Lasota M. (2020). Survival in Men Diagnosed with Prostate Cancer in Poland in the Years 2000–2014 Compared to European Countries Based on Concord-3. Rocz. Państw. Zakładu Hig..

[9] Grant W.B., Moukayed M. (2019). Vitamin D3 from Ultraviolet-B Exposure or Oral Intake in Relation to Cancer Incidence and Mortality. Curr. Nutr. Rep..

[10] Bouillon R., Antonio L., Olarte O.R. (2022). Calcifediol (25OH Vitamin D3) Deficiency: A Risk Factor from Early to Old Age. Nutrients.

[11] Sutherland J.P., Zhou A., Hyppönen E. (2022). Vitamin D Deficiency Increases Mortality Risk in the UK Biobank: A Nonlinear Mendelian Randomization Study. Ann. Intern. Med..

[12] Merchan B.B., Morcillo S., Martin-Nuñez G., Tinahones F.J., Macías-González M. (2017). The Role of Vitamin D and VDR in Carcinogenesis: Through Epidemiology and Basic Sciences. J. Steroid Biochem. Mol. Biol..

[13] Estébanez N., Gómez-Acebo I., Palazuelos C., Llorca J., Dierssen-Sotos T. (2018). Vitamin D Exposure and Risk of Breast Cancer: A Meta-Analysis. Sci. Rep..

[14] Pludowski P., Grant W.B., Bhattoa H.P., Bayer M., Povoroznyuk V., Rudenka E., Ramanau H., Varbiro S., Rudenka A., Karczmarewicz E. (2014). Vitamin D Status in Central Europe. Int. J. Endocrinol..

[15] Płudowski P., Ducki C., Konstantynowicz J., Jaworski M. (2016). Vitamin D Status in Poland. Pol. Arch. Intern. Med..

[16] Sewerynek E., Cieslak K., Janik M., Stuss M. (2014). Evaluation of Vitamin D Concentration in a Population of Young, Healthy Women: Effects of Vitamin D Supplementation. Endocr. Abstr..

[17] Rozmus D., Ciesielska A., Płomiński J., Grzybowski R., Fiedorowicz E., Kordulewska N., Savelkoul H., Kostyra E., Cieślińska A. (2020). Vitamin D Binding Protein (VDBP) and Its Gene Polymorphisms—The Risk of Malignant Tumors and Other Diseases. Int. J. Mol. Sci..

[18] Becker A.L., Carpenter E.L., Slominski A.T., Indra A.K. (2021). The Role of the Vitamin D Receptor in the Pathogenesis, Prognosis, and Treatment of Cutaneous Melanoma. Front. Oncol..

[19] Carlberg C., Velleuer E. (2022). Vitamin D and the Risk for Cancer: A Molecular Analysis. Biochem. Pharmacol..

[20] Trump D., Aragon-Ching J. (2018). Vitamin D in Prostate Cancer. Asian J. Androl..

[21] Łabędź N., Anisiewicz A., Stachowicz-Suhs M., Banach J., Kłopotowska D., Maciejczyk A., Gazińska P., Piotrowska A., Dzięgiel P., Matkowski R. (2024). Dual Effect of Vitamin D3 on Breast Cancer-Associated Fibroblasts. BMC Cancer.

[22] Carlberg C. (2022). Vitamin D and Its Target Genes. Nutrients.

[23] Klement R.J., Koebrunner P.S., Krage K., Sweeney R.A. (2021). Low Vitamin D Status in a Cancer Patient Population from Franconia, Germany. Complement. Med. Res..

[24] Płudowski P., Kos-Kudła B., Walczak M., Fal A., Zozulińska-Ziółkiewicz D., Sieroszewski P., Peregud-Pogorzelski J., Lauterbach R., Targowski T., Lewiński A. (2023). Guidelines for Preventing and Treating Vitamin D Deficiency: A 2023 Update in Poland. Nutrients.

[25] Wierzbicka E. (2022). Vitamin D Fortification in Dairy Products—Possibilities to Improve Vitamin D Intake. Postępy Tech. Przetwórstwa Spożywczego.

[26] Boparai J.K., Singh S., Kathuria P. (2019). How to Design and Validate A Questionnaire: A Guide. Curr. Clin. Pharmacol..

[27] Brzeziński J. (2003). Metodologia Badań Psychologicznych.

[28] Chandler P.D., Chen W.Y., Ajala O.N., Hazra A., Cook N., Bubes V., Lee I.-M., Giovannucci E.L., Willett W., Buring J.E. (2020). Effect of Vitamin D_3_ Supplements on Development of Advanced Cancer: A Secondary Analysis of the VITAL Randomized Clinical Trial. JAMA Netw. Open.

[29] Żórawska J., Szczepaniak W. (2024). The Problem of Increased Vitamin D_3_ Level in a Group of Patients Hospitalized in a Geriatrics Clinic. Med. Pracy.

[30] Rodd C., Sokoro A., Lix L.M., Thorlacius L., Moffatt M., Slater J., Bohm E. (2018). Increased Rates of 25-Hydroxy Vitamin D Testing: Dissecting a Modern Epidemic. Clin. Biochem..

[31] Muñoz A., Grant W.B. (2022). Vitamin D and Cancer: An Historical Overview of the Epidemiology and Mechanisms. Nutrients.

[32] Carlberg C., Muñoz A. (2022). An Update on Vitamin D Signaling and Cancer. Semin. Cancer Biol..

[33] Burt L.A., Billington E.O., Rose M.S., Raymond D.A., Hanley D.A., Boyd S.K. (2019). Effect of High-Dose Vitamin D Supplementation on Volumetric Bone Density and Bone Strength: A Randomized Clinical Trial. JAMA.

[34] Chang S.-W., Lee H.-C. (2019). Vitamin D and Health—The Missing Vitamin in Humans. Pediatr. Neonatol..

[35] Kuciński J., Fryska Z., Wołejko A., Semeniuk P., Burczyk R., Górna N., Łabuda A., Mazurek E. (2023). Current Recommendations in Poland for Vitamin D Supplementation. Med. Ogólna Nauki Zdrowiu.

[36] Zgliczyński W.S., Rostkowska O.M., Sarecka-Hujar B. (2021). Vitamin D Knowledge, Attitudes and Practices of Polish Medical Doctors. Nutrients.

[37] Fallon E.L., Lanham-New S.A., Williams P., Ray S. (2020). An Investigation of the Vitamin D Knowledge, Attitudes and Practice of UK Practising Doctors and Nurses: The D-KAP Study. Proc. Nutr. Soc..

[38] Saeed A., Eid M., Ahmed S., Abboud M., Sami B. (2020). Knowledge, Attitude, and Practice Regarding Vitamin D Deficiency among Community Pharmacists and Prescribing Doctors in Khartoum City, Sudan, 2020. Matrix Sci. Pharma.

[39] Safdar O., Baajlan O., Alamri A., Dahmash R., Alloush A., Ateeq R., Albokhari S., Zaher Z.F., Alghamdi M., Jiffri H. (2019). Assessment of Knowledge and Awareness of Vitamin D among Physicians and Students of Healthcare. Australas. Med. J..

[40] Agarwal A., Shah A., Shah B., Koottappillil B., Hirsch A.E. (2018). The Impact of a Radiation Oncologist Led Oncology Curriculum on Medical Student Knowledge. J. Cancer Educ..

[41] Matusitz J., Spear J. (2014). Effective Doctor–Patient Communication: An Updated Examination. Soc. Work Public Health.

[42] Ianovici C., Purcărea V.L., Gheorghe I.-R., Blidaru A. (2023). The Complexity of Physician-Patient Communication and Its Impact in Non-Medical Fields. A Surgical Oncology Approach. J. Med. Life.

[43] Löffler-Stastka H., Seitz T., Billeth S., Pastner B., Preusche I., Seidman C. (2016). Significance of Gender in the Attitude towards Doctor-Patient Communication in Medical Students and Physicians. Wien. Klin. Wochenschr..

[44] Tavakoly Sany S.B., Behzhad F., Ferns G., Peyman N. (2020). Communication Skills Training for Physicians Improves Health Literacy and Medical Outcomes among Patients with Hypertension: A Randomized Controlled Trial. BMC Health Serv. Res..

[45] Cohen P.A. (2016). The Supplement Paradox: Negligible Benefits, Robust Consumption. JAMA.

[46] Karbownik M.S., Paul E., Nowicka M., Nowicka Z., Kowalczyk R.P., Kowalczyk E., Pietras T. (2019). Knowledge about Dietary Supplements and Trust in Advertising Them: Development and Validation of the Questionnaires and Preliminary Results of the Association between the Constructs. PLoS ONE.

[47] Mozaffarian D., Rosenberg I., Uauy R. (2018). History of Modern Nutrition Science—Implications for Current Research, Dietary Guidelines, and Food Policy. BMJ.

[48] Guan T., Santacroce S.J., Chen D., Song L. (2020). Illness Uncertainty, Coping, and Quality of Life among Patients with Prostate Cancer. Psycho-Oncology.

[49] Ghodraty Jabloo V., Alibhai S., Fitch M., Tourangeau A., Ayala A.P., Puts M. (2017). Antecedents and Outcomes of Uncertainty in Older Adults with Cancer: A Scoping Review of the Literature. Oncol. Nurs. Forum.

[50] Kasprzycka K., Kurzawa M., Kucharz M., Godawska M., Oleksa M., Stawowy M., Slupinska-Borowka K., Sznek W., Gisterek I., Boratyn-Nowicka A. (2022). Complementary and Alternative Medicine Use in Hospitalized Cancer Patients—Study from Silesia, Poland. Int. J. Environ. Res. Public Health.

[51] Abuelgasim K.A., Alsharhan Y., Alenzi T., Alhazzani A., Ali Y.Z., Jazieh A.R. (2018). The Use of Complementary and Alternative Medicine by Patients with Cancer: A Cross-Sectional Survey in Saudi Arabia. BMC Complement. Altern. Med..

[52] Sirico F., Miressi S., Castaldo C., Spera R., Montagnani S., Di Meglio F., Nurzynska D. (2018). Habits and Beliefs Related to Food Supplements: Results of a Survey among Italian Students of Different Education Fields and Levels. PLoS ONE.

[53] Buckner C.A., Lafrenie R.M., Dénommée J.A., Caswell J.M., Want D.A. (2018). Complementary and Alternative Medicine Use in Patients Before and After a Cancer Diagnosis. Curr. Oncol..

[54] Gras M., Vallard A., Brosse C., Beneton A., Sotton S., Guyotat D., Fournel P., Daguenet E., Magné N., Morisson S. (2019). Use of Complementary and Alternative Medicines among Cancer Patients: A Single-Center Study. Oncology.

[55] Wojtacki J., Pawlowski L., Pawlowska I., Lichodziejewska-Niemierko M. (2017). Complementary and Alternative Medicine (CAM) Use among Patients with Cancer Undergoing Palliative Care: A Pilot Study of a Single Institution in Poland. J. Clin. Oncol..

[56] Hou Y.-N., Deng G., Mao J.J. (2019). Practical Application of “About Herbs” Website: Herbs and Dietary Supplement Use in Oncology Settings. Cancer J..

[57] Berretta M., Pepa C.D., Tralongo P., Fulvi A., Martellotta F., Lleshi A., Nasti G., Fisichella R., Romano C., De Divitiis C. (2017). Use of Complementary and Alternative Medicine (CAM) in Cancer Patients: An Italian Multicenter Survey. Oncotarget.

[58] Brooks S.L., Rowan G., Michael M. (2018). Potential Issues with Complementary Medicines Commonly Used in the Cancer Population: A Retrospective Review of a Tertiary Cancer Center’s Experience. Asia Pac. J. Clin. Oncol..

[59] Johnson S.B., Park H.S., Gross C.P., Yu J.B. (2018). Complementary Medicine, Refusal of Conventional Cancer Therapy, and Survival Among Patients With Curable Cancers. JAMA Oncol..

[60] Foley H., Steel A., Cramer H., Wardle J., Adams J. (2019). Disclosure of Complementary Medicine Use to Medical Providers: A Systematic Review and Meta-Analysis. Sci. Rep..

[61] Afzal N., Merchant A.A.H., Shaikh N.Q., Noorali A.A., Ahmad R., Ahmed S., Khan A.A., Bakhshi S.K., Abdul Rahim K., Mahmood S.B.Z. (2024). Patient-Resident Physician Communication—A Qualitative Study to Assess the Current State, Challenges and Possible Solutions. BMC Health Serv. Res..

[62] Little P., White P., Kelly J., Everitt H., Mercer S. (2015). Randomised Controlled Trial of a Brief Intervention Targeting Predominantly Non-Verbal Communication in General Practice Consultations. Br. J. Gen. Pract..

[63] Zhan Y., Mao P., Gao F., Shi Q. (2024). Content and Duration of Doctor-Patient Communication in Outpatient Oncology Follow-Up Consultations in China. Cureus.

[64] Al-Zahrani B.S., Al-Misfer M.F., Al-Hazmi A.M. (2015). Knowledge, Attitude, Practice and Barriers of Effective Communication Skills during Medical Consultation among General Practitioners National Guard Primary Health Care Center, Riyadh, Saudi Arabia. World Fam. Med. J. Middle East J. Fam. Med..

[65] Albahri A.H., Abushibs A.S., Abushibs N.S. (2018). Barriers to Effective Communication between Family Physicians and Patients in Walk-in Centre Setting in Dubai: A Cross-Sectional Survey. BMC Health Serv. Res..

