# Supplementary material for: Vitamin D-Related Information Exposure, Attitudes, and Practices Among Prostate and Breast Cancer Survivors in Poland: Implications for Patient–Physician Communication
Source: Nutrients. 2026 Jan 28;18(3):427. doi: 10.3390/nu18030427 (PMC12899719; doi:10.3390/nu18030427)
Supplement: Supplementary file 1 [file nutrients-18-00427-s001.zip › nutrients-4106155-supplementary.pdf]

*This survey questionnaire is anonymous. Participation in the study is voluntary. The responses obtained will provide information for a scientific publication* on attitudes and practices associated with vitamin D among individuals diagnosed with breast or prostate cancer. *The term "oncology care physician" used in the survey refers to any physician involved in the care of a patient diagnosed with cancer, e.g., a urologist, radiotherapist, surgical oncologist, etc. The organisers thank you for your willingness to participate in the survey.*

Questionnaire

## ELEMENTARY QUESTIONS

1. Have you heard claims that vitamin D deficiency may increase the risk of cancer?
 - ☐ Yes
 - ☐ No

2. Have you heard claims that abnormal vitamin D levels in the body can affect the course of cancer?
 - ☐ Yes
 - ☐ No

3. Have you been supplementing vitamin D since your cancer diagnosis?
 - ☐ No
 - ☐ Yes, and in connection with vitamin D supplementation, I have not discontinued any recommended therapeutic treatment
 - ☐ Yes, and in connection with vitamin D supplementation, I have discontinued recommended therapeutic treatments, recognising that vitamin D was more efficient in the treatment of my cancer than the medical recommendations

*If you have answered "no" to question 3, please skip questions 4 and 5.*

4. What dose of vitamin D have you been taking since your cancer diagnosis?
 - ☐ 1000 IU
 - ☐ 2000 IU
 - ☐ 4000 IU
 - ☐ other, …...................... IU

5. Does your oncology care physician know that you take vitamin D? *(you may tick more than one answer)*
 - ☐ Yes, I have informed the physician about it, and he/she has not forbidden me from using vitamin D
 - ☐ Yes, I have informed the physician about it, and he/she has forbidden me from using vitamin D
 - ☐ Yes, the physician knows about it, because he/she has ordered me to take vitamin D her/himself
 - ☐ Yes, the physician knows about it, because he/she has asked me whether I take vitamin D, and has not forbidden me from continuing to use it
 - ☐ Yes, the physician knows about it, because he/she has asked me whether I take vitamin D, and has forbidden me from continuing to use it
 - ☐ No, I have not informed the physician about it, because he/she has not asked me about it
 - ☐ No, I have not informed the physician about it, because I have forgotten to tell him/her about it
 - ☐ No, I have not informed the physician about it, because I considered it to be irrelevant

- ☐ No, I have not informed the physician about it, because I thought he/she might ridicule me
- ☐ No, I have not informed the physician about it, because I thought he/she might forbid me from using it
- ☐ Yes, because ……………………………………
- ☐ No, because ……………………………………

6. Have you used/are you using supplements other than vitamin D in connection with your cancer, e.g., herb-, mushroom-, amygdalin- or vitamin C-based supplements?

- ☐ No
- ☐ Yes, and I in connection with their supplementation, I have not discontinued any recommended therapeutic treatment
- ☐ Yes, and in connection with their supplementation, I have discontinued the recommended therapeutic treatment, recognising that supplements are more effective in the treatment of my cancer than the medical recommendations

*If you have answered "no" to question 6, please skip question 7.*

7. Does your oncology care physician know that you take supplements other than vitamin D? *(you may tick more than one answer)*

- ☐ Yes, I have informed the physician about it, and he/she has not forbidden me from using supplements
- ☐ Yes, I have informed the physician about it, and he/she has forbidden me from using supplements
- ☐ Yes, the physician knows about it, because he/she has ordered me to take supplements
- ☐ Yes, the physician knows about it, because he/she has asked me whether I take supplements, and has not forbidden me from continuing to use them
- ☐ Yes, the physician knows about it, because he/she has asked me whether I take supplements, and has forbidden me from continuing to use them
- ☐ No, I have not informed the physician about it, because he/she has not asked me about it
- ☐ No, I have not informed the physician about it, because I have forgotten to tell him/her about it
- ☐ No, I have not informed the physician about it, because I considered it to be irrelevant
- ☐ No, I have not informed the physician about it, because I thought he/she might ridicule me
- ☐ No, I have not informed the physician about it, because I thought he/she might forbid me from using these supplements
- ☐ Yes, because ……………………………………
- ☐ No, because ……………………………………

8. Who has recommended vitamin D supplementation to you since your cancer diagnosis?

- ☐ No one
- ☐ I have recommended it to myself
- ☐ A physician (please specify his/her specialisation ……………………………………………)
- ☐ A family member or an acquaintance who is not a physician

9. Who has recommended that you have your vitamin D levels tested since your cancer diagnosis?

- ☐ No one
- ☐ I have recommended it to myself
- ☐ A physician (please specify his/her specialisation ……………………………………………)
- ☐ A family member or an acquaintance who is not a physician

10. Do you think that your oncology care physician should be informed about your use of vitamin D and/or supplements based on other vitamins, herbs or mushrooms in connection with your cancer?
- ☐ Yes
- ☐ No
- ☐ It is difficult to answer “yes” or “no” because it depends on the physician’s views on this type of supplementation, and if one senses that the physician is not in favour of supplementation, it is better not to mention it

11. Do you perceive a need for physicians to routinely order tests for vitamin D deficiency in patients diagnosed with cancer?
- ☐ Yes
- ☐ No
- ☐ I have no opinion on this matter

## SOCIO-DEMOGRAPHIC DATA

1. Sex
- ☐ Woman
- ☐ Man

2. Age ………… years old

3. Educational background:
- ☐ Primary
- ☐ Basic vocational
- ☐ Secondary
- ☐ Higher

4. Place of residence
- ☐ Village
- ☐ City with a population of up to 100,000
- ☐ City with a population of over 100,000

5. Professional activity /you may tick more than one answer/
- ☐ Student
- ☐ I am still professionally active
- ☐ At present, on sick leave or receiving rehabilitation allowance
- ☐ Unemployed
- ☐ Retired employee
- ☐ Disability pensioner

6. Marital status
- ☐ Married
- ☐ Bachelor/maiden
- ☐ Divorced
- ☐ Widower/widow

7. Diagnosis of cancer(s) and approximate data of its/their diagnosis with an indication of whether it is a recurrence or metastatic disease /you may tick more than one answer/

- ☐ Prostate cancer – date of diagnosis: month…….…, year…………., ………………
- ☐ Breast cancer – date of diagnosis: month………, year…………., ………………
- ☐ ……………………– date of diagnosis: month………, year…………., ………………
- ☐ ……………………– date of diagnosis: month………, year…………., ………………

*If more than one answer was ticked in question 7, question 8 refers to the most recent cancer disease.*

8. Treatment received due to cancer /you may tick more than one answer/

- ☐ Surgery
- ☐ Radiotherapy
- ☐ Chemotherapy
- ☐ Hormone therapy
- ☐ Immunotherapy
- ☐ ………………………………
